# Evaluating the between-day reliability and likelihood of change of a test battery incorporating vastus lateralis muscle thickness, ankle-brachial pressure index, maximal voluntary torque, and six-minute walk test in patients with claudication

**DOI:** 10.1177/17085381241257735

**Published:** 2024-05-28

**Authors:** Thomas Parkington, David Broom, Thomas Maden-Wilkinson, Shah Nawaz, Markos Klonizakis

**Affiliations:** 1 Physical Activity, Wellness and Public Health Research Group, School of Sport and Physical Activity, 7314 Sheffield Hallam University, Sheffield, UK; 2 Centre for Physical Activity, Sport and Exercise Sciences, 2706 Coventry University, Coventry, UK; 3 Sheffield Vascular Institute, 7318 Sheffield Teaching Hospitals NHS Foundation Trust, Sheffield, UK; 4 Lifestyle, Exercise and Nutrition Improvement Research Group, Department of Nursing and Midwifery, 7314 Sheffield Hallam University, Sheffield, UK

**Keywords:** Peripheral artery disease, Six-minute walk test, Ankle-brachial index, maximal voluntary torque, muscle thickness

## Abstract

**Objective:**

The study aims to evaluate the between-day reliability of a proposed test battery for patients with claudication that can be used for monitoring the effectiveness of exercise interventions and other therapeutic strategies tailored to this patient population.

**Methods:**

Twenty-five men with claudication were recruited. The test battery consisted of the Vastus Lateralis muscle thickness (VL-MT), ankle-brachial pressure index (ABI), unilateral isometric knee extension maximal voluntary torque (MVT) and 6-minute walk test (6MWT). A single investigator conducted the tests for each patient on two separate testing sessions (T1 and T2) 5–7 days apart.

**Results:**

Good to excellent reliability was observed for VL-MT (ICC = 0.95, 95% LOA = ±3.10 mm, SEM = 0.81 mm), ABI (ICC = 0.97, 95% LOA = ±0.10, SEM = 0.02), MVT (ICC = 0.97, 95% LOA = ±24.0 N·m, SEM = 6.31 N·m), 6MWT distance (ICC = 0.99, 95% LOA = ±39.6 m, SEM = 11.0 m), 6MWT time to claudication (ICC = 0.99, 95% LOA = ±30.8 s, SEM = 7.8 s), and 6MWT ratings of pain (ICC = 0.87, 95% LOA = ±2.4 CR-10^+^, SEM = 0.7 CR-10^+^ ). Analysis derived from reliability data indicates a change of 1.4 mm for VL-MT, 0.14 for ABI, 12 N·m for MVT, 25 m for 6MWT distance, 15 s for 6MWT time to claudication and 1 CR-10^+^ for 6MWT ratings of pain is required to be interpreted as the minimum ‘likely’ change (76% chance).

**Conclusions:**

The test battery provides a reliable assessment of patients with claudication and can be widely used to evaluate the effects of exercise programmes and other therapeutic interventions. For the individual, changes in VL-MT, ABI, MVT, and 6MWT greater than the minimum likely change as a result of an exercise programme or an intervention are likely changes and less influenced by error associated with the test.

## Introduction

Claudication, a common and debilitating manifestation of peripheral artery disease (PAD), markedly impairs patients’ mobility and quality of life by inducing pain in the lower extremities during physical activity.^1^ Claudication occurs when the peripheral circulation is inadequate to meet the metabolic demand of the activity.^2^ National guidelines recommend exercise for the management of claudication, with strong evidence demonstrating improvement in functional capacity and alleviation of exertional leg symptoms.^3,4^

The multifaceted nature of claudication, influenced by both vascular insufficiency and musculoskeletal limitations, necessitates the use of comprehensive assessment tools.^5^ These tools must accurately evaluate the condition’s complex presentation to guide effective exercise strategies. In response to this need, a test battery combining measurements of vastus lateralis muscle thickness (VL-MT), ankle-brachial pressure index (ABI), isometric 90° knee extension maximal voluntary torque (MVT), and the six-minute walk test (6MWT) has been proposed. Each component of the test battery was selected for its direct relevance to claudication, addressing both the muscular and circulatory components of the disease. VL-MT is indicative of musculoskeletal health and muscle atrophy associated with PAD,^6^ ABI serves as a standardised measure of arterial sufficiency in PAD,^7^ MVT assesses strength and the potential for muscle weakness also associated with PAD,^8^ and 6MWT is a widely accepted measure of functional capacity in PAD.^9^

Despite the promising utility of this test battery, the between-day reliability of these measurements and their ability to detect clinically meaningful changes in patients with claudication remain underexplored. Therefore, this study aims to evaluate the between-day reliability of the test battery, comprising measurements VL-MT, ABI, MVT, and 6MWT, in patients with claudication. By establishing the reliability of these measurements, the study will assess the suitability of the test battery for monitoring the effectiveness of exercise interventions and other therapeutic strategies tailored to this patient population. Additionally, the study will determine the likelihood of positive, negative, or trivial changes in the measurements, which is essential for interpreting individual outcomes of therapeutic interventions, thereby guiding decision-making, and enhancing patient management.

## Methods

### Ethics

This study was part of a randomised controlled feasibility trial that explored the effect of low-intensity resistance exercise with blood flow restriction in patients with claudication.^10^ The study was approved by Yorkshire and The Humber – Leeds West Research Ethics Committee (REC reference: 20/YH/0039; IRAS project ID: 260419) and was conducted in accordance with The Code of Ethics of the World Medical Association (Declaration of Helsinki).

### Recruitment

Patients were recruited from the claudication clinics by Sheffield Vascular Institute of Sheffield Teaching Hospitals NHS Foundation Trust. Patients were screened by the clinical team for eligibility as part of a routing clinical assessment. Eligibility criteria included diagnosed PAD and ABI <0.9. Exclusion criteria included; unsuitable for or unable to exercise determined by study physician (SN), ABI >0.89, symptomatic presentation of rest pain, skin ulcers or gangrene and, impaired walking by a non-PAD condition or cannot walk without a walking aid. All patients were informed of the procedures and associated risks of the study before giving written informed consent.

### Study design

A single trained and experienced investigator (TP) conducted all the tests for each patient using standard operating procedures on two separate testing sessions (T1 and T2). The tests were performed in a systematic order: (1) VL-MT, (2) ABI, (3) MVT, and (4) 6MWT. Order was chosen in attempt to minimise the effects of the previous test influencing the subsequent test. The testing sessions were conducted at the same time of day for each patient, with T1 and T2 separated by 5 to 7 days.

### Vastus lateralis muscle thickness

**Figure 1.**
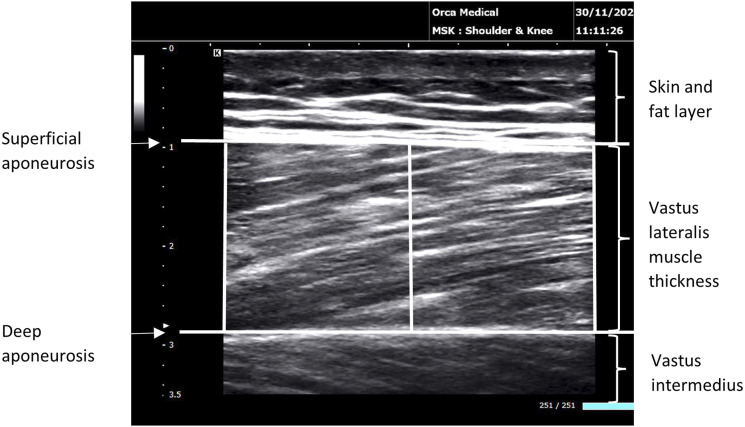
Representative recording of VL-MT at three different levels of the picture.

VL-MT was assessed via B-mode ultrasonography in accordance with standardised procedures.^11^ Both legs of the patient were measured at T1 and T2. Patients were supine on a hospital bed in a resting condition with the knee fully extended. The investigator waited 15 min before VL-MT measurement to allow fluid shifts to occur. The midpoint between the greater trochanter and lateral epicondyle and the midpoint between the medial and lateral edges of the vastus lateralis provided the reference point for ultrasound imaging. A 14 MHz probe (L14-4, Sonimage MX1, Konica Minolta, Tokyo, Japan) was longitudinally oriented at the reference point. A clear image of the superficial and deep aponeurosis was obtained and recorded. All images were analysed using computer software (ImageJ, U.S. National Institutes of Health, Maryland, USA). VL-MT was determined as the mean distance between the superficial aponeurosis and the deep aponeurosis at three different positions (left, middle, right) of the picture (Figure 1).

### Ankle-brachial pressure index

ABI was assessed using the standard Doppler ultrasound technique.^7^ A pneumatic cuff was placed around the upper arm with the lower edge of the cuff approximately 2 cm above the antecubital fossa and around the lower leg with the lower edge of the cuff approximately 2–4 cm above the ankle medial malleolus. A handheld vascular Doppler device (HI-Dop vascular Doppler, Ana Wiz, Surrey, UK) was positioned over the brachial, posterior tibial and dorsalis pedis arteries. The pneumatic cuff was inflated until Doppler flow signal was lost and the systolic blood pressure was determined by the first Doppler flow signal while deflating the cuff from a suprasystolic level. Systolic blood pressure was measured sequentially for the brachial, dorsalis pedis and posterior tibial arteries. The ABI was calculated by dividing the higher of the dorsalis pedis or posterior tibial pressure of each leg by the higher of the right or left brachial artery pressure. ABI was measured for each leg and values <0.9 were used for analysis.

### Maximal voluntary torque

Unilateral isometric knee extension MVT was assessed using an isokinetic dynamometer (Cybex Humac Norm Isokinetic Extremity System, Computer Sports Medicine Incorporated, Massachusetts, USA). Patients were seated with their knee and hip joints positioned at 90° flexion. The dynamometers axis was aligned with the knee joint, and the lower leg was secured to the lever arm. Straps stabilised the thigh, pelvis, and torso to minimised extraneous movements.

Patients warmed up by performing five repetitions of isometric knee extensions held for 3 seconds at submaximal effort. For the maximal effort test, patients performed three repetitions of isometric knee extensions held for 3 seconds at maximal effort. At least 1 minute of rest was provided between efforts to prevent fatigue. Visual feedback of the force signal and strong verbal encouragement were provided during testing to motivate maximal efforts. The highest torque value recorded was used for analysis.

### Six-minute walk test

The 6MWT was performed following the American Thoracic Society guidelines.^12^ The 6MWT was performed indoors, in a long, flat, and straight corridor on hard flooring with the walking circuit set at 20 m in length. Patients were seated at the starting position for 5 min before starting the test to ensure the recovery of the previous activity. Patients were asked to complete as many shuttles as possible in 6 min. Patients were informed that during the test they could rest at any point and continue again when ready. Total distance covered, time to claudication, and pain at end of the test assessed using the Borg CR-10^+^ scale^13^ was recorded and used for analysis.

### Data analysis

The between-day reliability of VL-MT, ABI, MVT and 6MWT was assessed through several statistical analyses following the guidelines of Atkinson and Nevill.^14^ Histogram plots of data distribution and Shapiro-wilk test of normality confirmed normal distribution of all measurements. Additionally, the mean difference between T1 and T2 followed a normal distribution for all measurements. Systematic bias between T1 and T2 was assessed via a paired *t*-test for each measurement. Statistical significance was set at *p* ≤ .05. Absolute reliability was assessed by standard error of measurement (SEM) and 95% limits of agreement (95% LOA). Relative reliability was assessed by intraclass correlation coefficients (ICC) with 95% confidence intervals. Bland–Altman plots^15^ were compiled to present systematic and random error trends for all measurements.

To provide a practical context to reliability data, we present measurement changes to interpret the likelihood of change using the analysis approach of Hopkins.^16^ The analysis quantifies the probability (%) that a given measurement change is positive, trivial, and negative, in context to the measurement’s reliability. Specifically, a positive change is beneficial and indicates an improvement beyond the measurement error, a trivial change is within measurement error suggesting no real change, and a negative change represents a detrimental change and indicates a decrement in outcome. Furthermore, the analysis facilitated the identification of the minimum measurement change considered likely beneficial, set at a threshold with a 76% probability of being a true improvement. This threshold supports in distinguishing meaningful changes from those attributable to measurement variability.

## Results

Thirty patients were recruited as part of the randomised controlled feasibility study.^10^ Five patients opted to not take part in the reliability aspect of the study. In total, twenty-five older (69 ± 9 years), overweight (BMI = 26.6 ± 3.4, stature = 174 ± 7 cm, body mass = 81 ± 11 kg), symptomatic (44% bilateral claudication) men with moderately severe PAD (ABI = 0.68 ± 0.14) participated in this study. Twenty percent were current smokers, 64% were previous smokers and 16% had never smoked.

**Figure 2.**
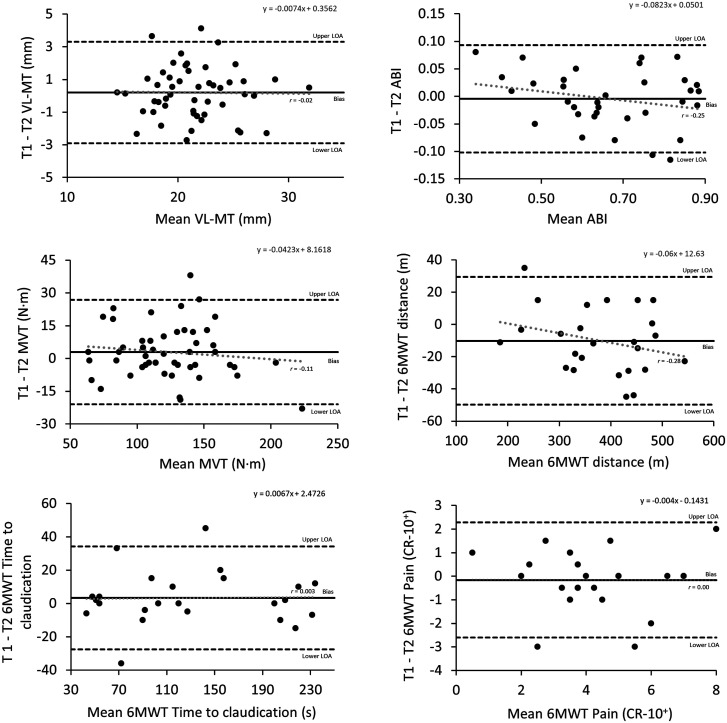
Bland-Altman plot of VL-MT, ABI, MVT and 6MWT distance. The solid line represents the bias and dashed line represents the 95% limits of agreement.

Table 1 presents the measurements values at T1 and T2. A small but significant systematic bias was evident for 6MWT distance with T2 higher than T1 (4%). Table 2 presents the between-day reliability of measurements. Good to excellent reliability was observed for all measurements with ICC of ≥0.87. Figure 2 displays Bland-Altman plots for measurements. Few observations were outside the LOA. Small proportional bias (*r* ≤ −0.28), though statistically insignificant (*p* > .05), indicated presence of heteroscedasticity for ABI, MVT, and 6MWT distance as the values for differences decreased as the mean values increased.

**Table 1.** Mean (±SD) of measurements at T1 and T2 and associated bias.

Variable	Test 1	Test 2	Bias ± SD	*p*
VL-MT (mm)	21.6 ± 3.6	21.4 ± 3.7	0.2 ± 1.6	.38
ABI	0.66 ± 0.15	0.67 ± 0.16	0.00 ± 0.05	.49
MVT (N·m)	125.0 ± 34.2	122.1 ± 35.6	2.9 ± 12.2	.10
6MWT				
Distance (m)	375.4 ± 91.1	385.6 ± 96.7	−10.2 ± 20.2	.02
Time to claudication (s)	127.9 ± 67.1	124.6 ± 66.7	3.3 ± 15.7	.30
Pain (CR-10^+^)	4.2 ± 1.8	4.3 ± 1.9	−0.2 ± 1.2	.53

**Table 2.** Between-day reliability of measurements.

Variable	95% LOA (±)	SEM	ICC
VL-MT (mm)	3.10	0.81	0.95 [0.91, 0.97]
ABI	0.10	0.02	0.97 [0.95, 0.99]
MVT (N·m)	24.0	6.31	0.97 [0.94, 0.98]
6MWT (m)			
Distance (m)	39.6	11.0	0.99 [0.96, 0.99]
Time to claudication (s)	30.8	7.8	0.99 [0.97, 0.99]
Pain (CR-10^+^)	2.4	0.7	0.87 [0.72, 0.95]

Table 3 presents the likelihood of changes in VL-MT, ABI, MVT and 6MWT using the approach of Hopkins.^16^ Three measurement changes are proposed for each variable and the analysis assessed the probability the ‘true change’ is positive, trivial, or negative. In addition, this analysis identified the minimum change in measurement because of treatment or intervention (e.g. supervised exercise programme) that is required to be interpreted as likely beneficial (Table 4).

**Table 3.** Likelihood of measurement changes.

Variable	Measurement change	Probability of change (%)		
		Negative	Trivial	Positive
VL-MT (mm)	1	23	8	69
	2	8	5	87
	3	2	2	96
ABI	0.10	0	50	50
	0.15	0	19	81
	0.20	0	4	96
MVT (N·m)	10	15	13	72
	20	3	5	92
	30	0	2	98
6MWT distance (m)	20	7	24	68
	40	1	7	92
	60	0	1	99
6MWT time to claudication (s)	20	8	7	85
	30	2	3	95
	40	1	1	98
6MWT pain (CR-10^+^)	1	20	5	76
	2	6	2	92
	3	1	1	98

**Table 4.** Measurement changes required to be interpreted as likely beneficial (76% chance).

Variable	Measurement change
VL-MT	1.4 mm
ABI	0.14
MVT	12 N·m
6MWT, distance	25 m
6MWT, time to claudication	15 s
6MWT, pain	−1 CR-10^+^

## Discussion

This study aimed to evaluate the reproducibility and determine minimal clinically significant changes within a test battery tailored for patients with claudication due to PAD. By establishing the between-day reliability of the measurements and quantifying the smallest changes that represent meaningful improvements, the test battery can be confidently used to monitor and guide interventions, particularly exercise programmes, tailored to this patient population. These findings extend from our previous work which explored the effects of low-intensity resistance exercise with blood flow restriction in patients with claudication.^10^ Unlike our previous work, which used the test battery to assess the efficacy of the novel exercise intervention, the current manuscript focuses on rigorously validating the reliability of these measures and establishes thresholds for meaningful changes. These developments are critical for the scientific validation of the test battery that were not addressed previously. Data presented in this paper underpin the robust use of these measures in clinical and research settings, which was beyond the scope of the previous work.

This study confirmed the excellent reliability for VL-MT with an ICC 0.95, aligning with previous findings in healthy older and young adults^17,18^ and patients with knee osteoarthritis.^19^ A minimal change of 1.5 mm in VL-MT was identified as the threshold required to detect a likely beneficial change (76% chance) in patients with claudication, with smaller changes potentially leading to interpretative uncertainties of the true effect following an intervention.

Assessing muscle morphology is crucial in claudication management due to the direct relationship between ischaemia, muscle size, and functional capacity.^20^ In PAD patients, reduced physical activity contributes significantly to the atrophy of lower extremity muscles^21^ and reperfusion injury can directly cause skeletal muscle damage, further exacerbating myopathy.^22^ Studies have shown that more adverse muscle characteristics correlate with increased rates of mobility loss and higher mortality, highlighting the importance of muscle assessment in this patient population.^23,24^

While the gastrocnemius muscle has traditionally been a focus in PAD assessments,^25^ the vastus lateralis offers several advantages. Its larger size may reflect systemic muscle health more accurately and facilitate comparisons of changes over time or in response to interventions.^26^ As a crucial part of the quadriceps group, the vastus lateralis plays a pivotal role in performance of activities such as walking and stair climbing, which are often impaired in PAD.^27^ Rehabilitation strategies are often focused on improving walking performance, balance, and daily functioning through targeted thigh muscle exercises.^28^ As such, the measurement of VL-MT is relevant for evaluating the efficacy of interventions.

In this study, ABI were obtained at rest using the gold standard Doppler ultrasound technique, demonstrating excellent reliability with an ICC of 0.96. This level of agreement aligns with prior studies across the various clinical stages of PAD (i.e. asymptomatic, claudication, rest pain, ulcers, and gangrene),^29,30^ indicating consistent reliability of ABI measurements. ABI measured at rest was used in this study over ABI following exercise due to its widespread acceptance and regular use in clinical evaluations of PAD.^31^ While exercise ABI is important in revealing functional impairments not apparent at rest, resting ABI is more feasible in a broader range of clinical settings, making it a practical choice for this initial reliability assessment.

In clinical practice, a change of >0.15 in ABI is generally accepted as clinically significant, provided there is supporting evidence of haemodynamic improvement or deterioration.^32^ To suggest a successful intervention, an increase of 0.10 in ABI has been recommended as an objective criterion.^33^ Our findings refine these thresholds, suggesting that increases of 0.08 and 0.14 in ABI indicate a likely beneficial change with a 76% certainty and a very likely beneficial change with greater than 95% certainty, respectively.

Assessing lower extremity muscle strength is vital in managing claudication due to its association with mobility, quality of life and mortality.^8^ The present study observed excellent reliability (ICC = 0.94) for MVT, comparable to findings in older adults.^34^ However, a trend towards significant bias (*p* = .06) was observed, suggesting that reliability could be improved with prior familiarisation. Our findings suggest that a change of 17 N·m and 30 N·m is required to detect a likely beneficial change with 76% certainty and a very likely change with over 95% certainty, respectively. Increasing the confidence in interpreting small changes in MVT would require improved reliability.

Walking performance is a focus of PAD assessment given the impairment associated with PAD and the limitations of poor walking performance has on patients’ quality of life.^9^ The 6MWT is a well-accepted assessment of walking performance and demonstrated excellent reliability in this study (ICC = 0.97) which is consistent with previous studies in patients with claudication,^35^ chronic heart failure,^36^ and knee osteoarthritis.^37^ Our observed 6MWT distance (382.9 ± 88.5 m) is similar to previous findings (397.9 ± 80.2 m) which measured a large sample of 100 patients with claudication.^35^ Notably, there was a significant systematic bias with a mean increase of 13.8 m in T2 (*p* = .03), likely due to a learning effect as previously reported in chronic heart failure.^36^ Practitioners should be aware of this potential bias when interpreting 6MWT distance in claudication patients.

The present study identified an increase of 35.5 m in 6MWT distance is required to detect a likely beneficial change (76% certainty) and increase over 60 m to detect a very likely change (>95% certainty). Prior to this study, the minimal detectable change of 6MWT test in patients with claudication was determined as 46 m.^35^ Additionally, the minimal clinically important difference in 6MWT distance, which represents the smallest threshold change in an outcome measure that is perceived beneficial to the patient has been reported in patients with claudication.^38^ After 3 months of exercise intervention, small (5%), moderate (25%), and large (40%) clinically important changes in the 6MWT distance were found to be at 12 m, 32 m, and 34 m, respectively.^38^

Time to claudication and pain ratings during the 6MWT are less reported than distance in the assessment of walking performance in PAD. Though, time to claudication and pain is an objective metric to assess the severity of claudication and it impact on walking performance and can provide greater insights into claudication’s effect on walking performance. The results indicate excellent reliability for time to claudication with ICC of 0.99 and good reliability for pain with ICC of 0.87 supporting its reporting in the 6MWT.

The proposed test battery is a reliable tool to assess patient’s physical condition and monitor for therapeutic interventions in claudication management. By providing a detailed assessment of both circulatory and muscular health, the test battery can support practitioners to personalise exercise and rehabilitation programmes to individual patient needs to potentially improve outcomes. Furthermore, the identification of smallest worthwhile change in measurements aids practitioners in interpreting changes over time, offering a nuanced understanding of patient progress.

A strength of this study is its comprehensive and practical approach to evaluating claudication, supported by rigorous statistical analysis of reliability and likelihood of change. A limitation of the study is the relatively small sample size and the study’s single-centre design, which may affect generalisability. A larger sample size would reduce the random error of measurement that can make the mean of the measurement different for each test as random errors of the measurement can cancel out when more data are included together for calculation of the mean.^39^ Furthermore, a larger sample would provide greater precision of estimates of ICC.^14^ Nevertheless, the reliability of VL-MT, ABI, MVT, and 6MWT presented here are comparable to previous studies.

In conclusion, this study affirms the good to excellent reliability of a test battery (VL-MT, ABI, MVT and 6MWT) in assessing PAD severity and the effectiveness of exercise programmes and other therapeutic interventions in patients with claudication. By providing thresholds for likelihood of changes, practitioners are equipped with critical tools for interpreting intervention outcomes.
